# A Systematic Review of the Efficacy of Early Initiation of Speech Therapy and Its Positive Impact on Autism Spectrum Disorder

**DOI:** 10.7759/cureus.35930

**Published:** 2023-03-09

**Authors:** Hafsa A Osman, Merna Haridi, Natalie A Gonzalez, Sana M Dayo, Umaima Fatima, Aaiyat Sheikh, Chaitanya S Puvvada, Faiza H Soomro, Safeera Khan

**Affiliations:** 1 Pediatrics, California Institute of Behavioral Neurosciences and Psychology, Fairfield, USA; 2 Medical Education, St. Martinus University, Willemstad, CUW; 3 Medical Education, California Institute of Behavioral Neurosciences and Psychology, Fairfield, USA; 4 Obstetrics and Gynecology, California Institute of Behavioral Neurosciences and Psychology, Fairfield, USA; 5 Public Health Sciences, Liaquat University of Medical and Health Sciences, Jamshoro, PAK; 6 Internal Medicine, California Institute of Behavioral Neurosciences and Psychology, Fairfield, USA; 7 Internal Medicine, Era's Lucknow Medical College and Hospital, Lucknow, IND; 8 General Surgery, California Institute of Behavioral Neurosciences and Psychology, Fairfield, USA; 9 General Surgery, Ninewells Hospital, National Health Service (NHS) Tayside, Dundee, GBR

**Keywords:** language and social skills, language disorder, speech therapy, early intervention, autism spectrum disorder (asd)

## Abstract

Autism spectrum disorder (ASD) is a condition that consists predominantly of an apparent early delay in communication and social skills. Among the multiple identified etiologies, genetics play a key role. The implementation of early interventional therapy for children with ASD is starting to show promising results. A few medical databases were used to collect multiple published types of research, which were thoroughly screened. Ultimately, a small amount was selected according to the defined eligibility criteria. The 12 articles that were reviewed involved a more significant number of boys than girls, and most clinical trials displayed the importance of starting early therapy. Astonishingly, the overwhelming effects of the COVID-19 pandemic did not affect the continuation of speech therapy in certain areas. In addition, studies emphasize knowledge scarcity, insufficient resources in certain areas, and the demand to educate the community. Conversely, no difference in the level of severity was noted with the implementation of early therapy. Early therapy, chiefly speech therapy used to treat children with ASD, demonstrated favorable outcomes. Communities require awareness about the condition on a broader scale to educate caregivers on early alarming symptoms. All in all, additional exploration needs to be done.

## Introduction and background

According to recent statistics, roughly one in every 44 children is diagnosed with autism spectrum disorder (ASD) worldwide. The median male-to-female ratio is 4:1, with prevalence estimates increasing drastically over time and showing a wide distribution among various ethnicities and children from multiple socioeconomic backgrounds [1].

ASD is a rising developmental disorder characterized by apparent early deficits in social communication, restricted interests, and repetitive behaviors that vary in severity [2]. ASD can be diagnosed clinically by several qualified professionals, including pediatricians, psychiatrists, or psychologists [3]. An update to the Diagnostic and Statistical Manual of Mental Disorders 5 (DSM-5) was published in 2013, updating the diagnostic criteria for diagnosing ASD. As a result, the notion of ASD diagnosis spectrum was created, combining the pervasive developmental disorder (PDD) diagnoses: autistic disorder, childhood disintegrative disorder, Asperger's syndrome, and finally, PDD not otherwise specified (PDD-NOS), all under the same umbrella. Rett syndrome is, therefore, considered a separate neurological disorder [2]. A condition that highly coexists with autism is attention-deficit hyperactivity disorder (ADHD) [3]. 

In light of recent research on autism, the following were identified etiologies, and an irrefutable known etiology is the role of genetics on ASD. With monozygotic (identical) twins, if one twin is autistic, the likelihood of the other twin having some form of autism is 90%. However, for dizygotic (fraternal) twins, the possibility of the other twin having some form of autism is as little as 2% to 3%. There are multiple debates regarding the association between vaccine administration and the development of autism. For instance, a hypothesis that was studied about the cause of autism was the pertussis toxin in the diphtheria, pertussis, and tetanus (DPT) vaccine causing a separation of the G-alpha protein from retinoid receptors in genetically susceptible children. A striking fact that is likely overlooked is how thimerosal, a commonly used yet unfavorable antibacterial agent in vaccines, is suspected to be a known cause [4-6].

With the clinical cases of autism substantially rising, clinical research on autism is increasing considerably. Research helps to unravel the unsolved questions that continue to linger and bring several advancements to what is currently known. Several genes have been studied within families to help identify the cause and are now being investigated [7]. Neuroimaging is a frequently used modality to study the brain structure of children with ASD, and an interesting finding was that both frontal and temporal lobes are affected [8]. Multiple drugs are currently used to treat autism, including antipsychotics, antidepressants, selective serotonin reuptake inhibitors (SSRIs), α2 and β-adrenergic antagonists, mood stabilizers, and anticonvulsants [9,10]. Implementing early screening using the Modified Checklist for Autism in Toddlers (M-CHAT) makes an immense difference in early diagnosis, thus improving the overall outcome [11].

On the contrary, there are still missing pieces to the puzzle regarding a thorough knowledge of ASD. Challenges remain in making a diagnosis and recognizing the core symptoms of ASD, along with limited resources and poor adherence to treatment options [12]. There is no doubt that there still exists a demand for additional research, along with implementing early behavioral programs to provide parents with easily accessible resources to help improve the lives of affected children [13]. The effect of the stigma placed on children with ASD can further widen the gap as communities are still not fully aware of the disorder, leading to additional confusion. This barrier can be overcome by providing communities with practical resources to help families develop an in-depth understanding and future expectations [14]. However, providing the best care possible will require all involved parties to come together, sharing a common goal. Just like the saying goes, *teamwork makes the dream work*.

This study aims to highlight the profound importance of early intervention, mainly speech therapy, and its beneficial impact on children with ASD, bringing about a significant change. Recent advancements have been made and new interventions are starting to bloom; nonetheless, clear gaps remain.

**Figure 1:**
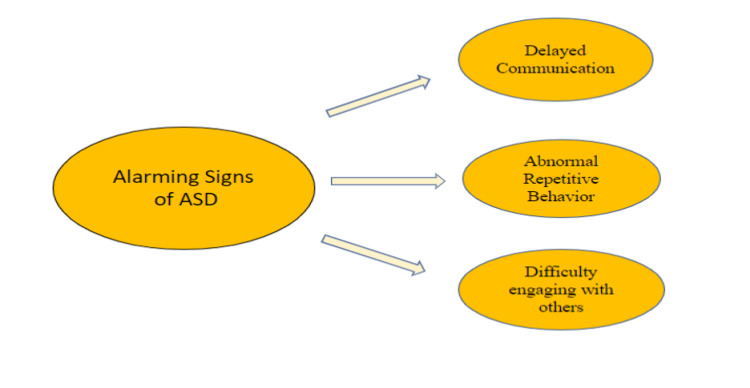
The alarming signs of autism spectrum disorder. Figure credits: All the authors. ASD, autism spectrum disorder

The alarming signs of ASD are shown in Figure 1.

## Review

Methods

This systematic review was conducted and the following data were reported using the Preferred Reporting Items for Systematic Reviews and Meta-analysis (PRISMA) 2020 guidelines [15].

Search sources and search strategy

The systematic review was conducted using the following medical databases: PubMed, PubMed Central (PMC), PubPsych, and Science Direct, which were conducted from September 18, 2022, to October 1, 2022. The keywords "autism spectrum disorder," "speech therapy," and "early intervention" were combined differently, generating a pool of published research. A slightly different approach was used for the PubMed database, which involved applying the Medical Subject Heading (MeSH) strategy with the help of the PubMed MeSH database. It involved linking "Autism Spectrum Disorder/diagnosis"[Mesh] OR "Autism Spectrum Disorder/genetics"[Mesh] AND "Early Intervention, Educational"[Mesh] OR "Autism Spectrum Disorder/diagnosis"[Mesh] OR "Autism Spectrum Disorder/genetics"[Mesh] OR "Autism Spectrum Disorder/physiopathology"[Mesh] AND "Speech Therapy"[Mesh]. This latter strategy was able to help target researches that best resembled the review topic.

Eligibility criteria

Inclusion and Exclusion Criteria 

The articles selected for this review were limited between 2007 and 2022. The articles chosen met the following criteria: the articles were written and published in the English language, included speech therapy as a form of intervention for children diagnosed with autism, and most importantly, the study subjects were children clinically diagnosed with ASD. The selection of documents was completed by October 1, 2022. The excluded articles included those which were not aligned with the targeted research goal, gray literature, and articles that were not full text and had missing abstracts. 

Results

Search Results

**Figure 2:**
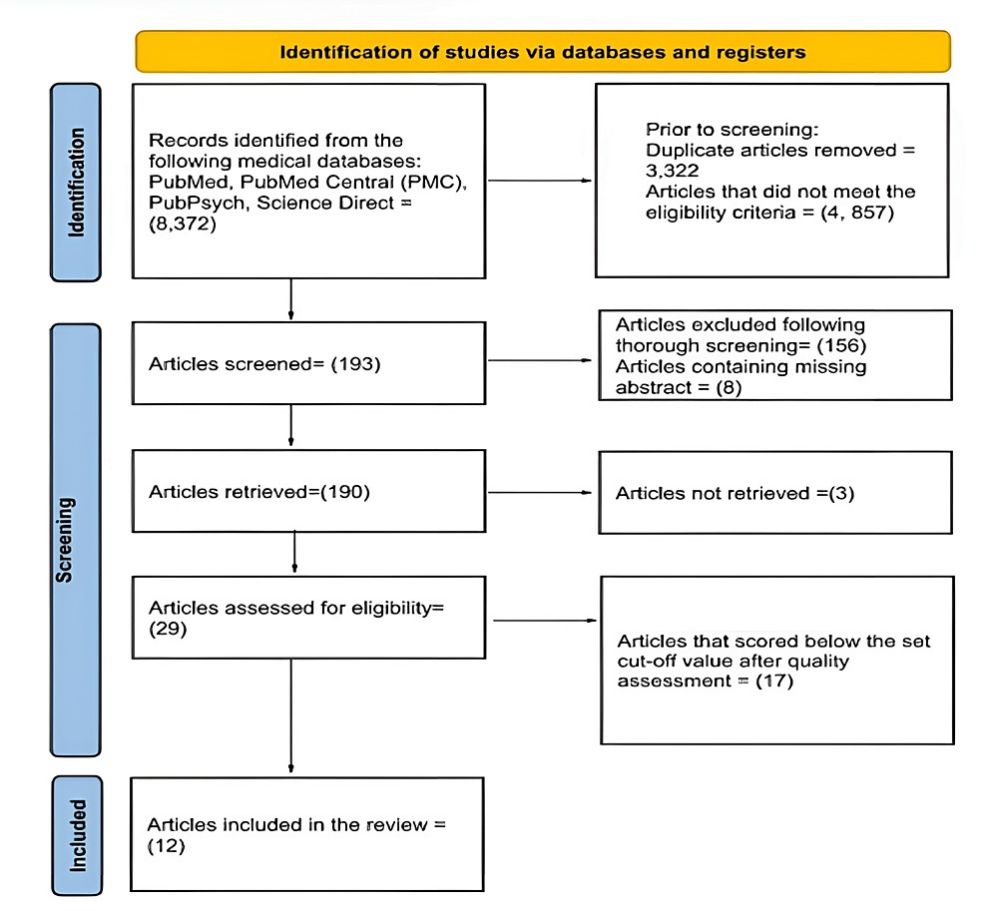
The search process flow using the PRISMA flowchart. PRISMA, Preferred Reporting Items for Systematic Reviews and Meta-Analyses

Out of 8,372 articles found during the search process, 4,857 were removed as they did not meet the inclusion criteria and 3, 322 were duplicates. During the screening process, all the articles were thoroughly read; 156 articles were further removed, while eight articles contained missing abstracts. A thorough quality assessment of the remaining articles was done, and 17 more articles were excluded due to the reports being below the set cutoff point concerning the quality of the papers. Finally, 12 articles remained and were included in the review. Figure 2 displays the search process flow using the PRISMA flowchart [15].

Quality Assessment of the Studies

The critical appraisal for the randomized clinical trials was assessed using the Cochrane Risk-of-Bias Assessment tool [16]. Furthermore, the nonrandomized clinical trials, observational studies, case reports, and quasi-experimental studies were all appraised using the Joanna Briggs Institute (JBI) tool [17]. Finally, two nonrandomized clinical trials were clinically appraised using the Newcastle Ottawa tool [18]. The cutoff value was set at 70%, and studies with a score of 70% or more were included in the systemic review.

Table 1 displays the JBI tool for the Cochrane Risk-of-Bias Assessment tool [16].

**Table 1:** Cochrane Risk-of-Bias Assessment tool.

Author name​ ​ ​	Random sequence allocation​ ​ ​	Allocation unknown​ ​ ​	Blinding of staff and participants​ ​ ​	Blinding of effect​ ​ ​	Thorough outcome data​ ​ ​	Selective reporting​ ​ ​	Additional sources of bias​ ​ ​
Malucelli et al. [19]​	Low risk	​Low risk	​Low risk	​Unclear risk	​Low risk	​Low risk	​Low risk
Boyd et al. [20]​	​Low risk	​Low risk	​Unclear risk	​High risk	​Low risk	​Low risk	​Low risk
Gepner et al. [21]​	​Low risk	​Low risk	​High risk	​High risk	​Low risk	​Low risk	​Low risk

Table 2 displays the JBI for a cross-sectional study [17].

**Table 2:** JBI tool for a cross-sectional study. JBI, Joanna Briggs Institute

Author name​ ​ ​	Inclusion criteria clearly defined​ ​ ​	Study subjects and setting clearly described​ ​ ​	Valid and reliable exposure​ ​ ​	Objective standard criteria​ ​ ​	Identified confounding factors​ ​ ​	Strategies dealing with confounding factors​ ​	Valid & reliable outcome​ ​ ​	Appropriate statistical analysis​ ​ ​
​ Alotaibi and Almalki [22]	​Low risk	​Low risk	​Low risk	​High risk	​Unclear risk	​Low risk	​Low risk	​Low risk

Table 3 displays the JBI tool for a quasi-experimental study [17].

**Table 3:** JBI tool for a quasi-experimental study. JBI, Joanna Briggs Institute

Author name​ ​ ​	Clear cause and effect​ ​ ​	Comparing participants similarly​ ​ ​	Similar treatment in comparison group​ ​ ​	Control group included​ ​ ​	Multiple outcomes for both pre- and postintervention​ ​ ​	Complete follow-up​ ​ ​	Measured outcome of the comparison group​ ​ ​	Reliable outcome measure​ ​ ​	Appropriate statistical analysis​ ​ ​
​ Chamalah and Arsanti [23]	​Low risk	​Low risk	​Low risk	​High risk	​Low risk	​Low risk	​High risk	​Low risk	​Low risk

Table 4 displays the New Castle Ottawa tool [18].

**Table 4:** Newcastle Ottawa Scale. 1a*, representativeness of a cohort study; 1b*, selection of non-cohort subjects; 1c*, ascertainment of exposure; 1d*, outcome of interest available at the beginning of the study; 2*, comparability of cohorts based on study design or analysis; 3a*, assessment of the study outcome; 3b*, appropriate follow-up of cohorts.

Author name​	1a*: Selection ​	1b*: Selection ​	1c*: Selection​	1d*: Selection​	2*: Comparability​	3a*: Outcome​	3b*: Outcome​
​ Zhou et al. [24]	​Yes	Yes​	​Yes	Yes​	​Yes	​Yes	Yes​
​ Rollins et al. [25]	​Yes	​Yes	​Yes	​No	​Yes	​Yes	Yes​
​ Osborne et al. [26]	​Yes	Yes​	​Yes	​No	Yes​	​Yes	​Yes
​ Nair et al. [27]	​Yes	​Yes	​Yes	​Yes	​Yes	​No	​No

Table 5 displays the JBI tool for qualitative research [17].

**Table 5:** JBI tool for qualitative research. JBI, Joanna Briggs Institute

Author name​ ​ ​	Congruity between stated perspective and methodology​ ​ ​	Congruity between methodology and objective​ ​ ​	Congruity between methodology and data collection method​ ​	Congruity between methodology and data analysis​ ​ ​	Congruity between methodology and data interpretation​ ​ ​	Statement on researcher’s cultural belief & values​ ​ ​	Any influence on research​ ​ ​	Representation of participants voices​ ​ ​	Evidence on ethical appropriateness ​ ​ ​	Relationships between conclusions drawn & interpretation of data​ ​ ​
​ Karrim et al. [28]	​Low risk	​Low risk	​Low risk	​Low risk	​Low risk	​Low risk	​Low risk	​Low risk	​Low risk	​Low risk

Table 6 displays the JBI tool for a case report [17].

**Table 6:** JBI tool for a case report. JBI, Joanna Briggs Institute

Author name​ ​ ​	Demographic characteristics clearly defined​ ​ ​	Clear history and presentation of patient timeline​ ​ ​	Clear description of patient condition​ ​ ​	Clearly described diagnostic test and assessment method​ ​ ​	Clearly described diagnostic test and assessment method​ ​ ​	Postintervention clearly described​ ​ ​	Any adverse or unanticipated events​ ​ ​	Takeaway lessons from the case report​ ​ ​
​Wannenburg and van Niekerk [29]	​Low risk	​Low risk	​Low risk	​High risk	​Low risk	​Low risk	​Unclear risk	​Low risk

Study Characteristics 

More than half of the studies included in this review are clinical trials conducted over a one-month to four-year period. The greater number of participants included in the study were children who were clinically evaluated and ultimately diagnosed by a qualified professional. The study population comprised 262 boys and 72 girls, while three out of 12 studies did not specify the gender. This previous statement supports autism being four times more common in males than in females, which is still a ratio that continues to persist [1]. Meanwhile, the articles included mainly focused on the positive outcomes that children with autism continue to benefit from specifically in their language and social skills.

Furthermore, three articles emphasized the negative consequences of stress on caregivers and how combining the right resources and guidance from trusted institutions can significantly reduce it. A surprisingly interesting article about telerehabilitation services offered to six children with autism drew attention to how interventions can still be possible even during difficult times like the deadly COVID-19 pandemic. Finally, a common and widely used intervention in almost all of the studies was speech therapy, which showed tremendous improvement in language skills.

Study Results 

The study articles contained a total of 501 participants, while one study did not include the number of participants involved. The studies that specified gender comprised 78% males and 22% females. The primary outcome assessed was the effect of starting intervention early on, which showed promising results. The key improvements reported were a substantial increase in cognitive ability, effective communication, better social skills, and, most importantly, a decrease in anxiety levels. Another notable outcome was that children with ASD could better express themselves, improving their self-esteem [29,30]. Three articles reported no association between early intervention and the severity of autism [26,27,30]. However, parental education remains crucial in providing essential resources to all caregivers to start treatment as soon as red flags are spotted. In light of this statement, several articles show the importance of educating parents. This can help parents be aware of the diagnosis in addition to treatment options to have a more vital understanding of the potential obstacles they may face [19,22,25,26,27,30]. Unfortunately, a lack of awareness intertwined with a lack of resources can considerably increase the stress levels of caregivers. The preceding statement can be supported by articles demonstrating the distressing effects of stress on caregivers [20,26,27]. Finally, the value of a sound support system can be appreciated by the results of four articles on how it helps children with autism thrive within their own space [25,26,28,29]. Table 7 displays the summary of the study results.

**Table 7:** The summary of the study results. ASD, autism spectrum disorder; ESDM, Early Start Denver Model

Name of author​	Year of publication​	Aim of study​	Study method​	Main outcome of the study​
Alotaibi and Almalki [22]	2016​	Parental perception of early intervention​	Observational study​	Scarcity of resources for early intervention.​
Clark et al. [30]	2018​	Comparing outcomes of children depending on the time of diagnosis​	Cohort study​	Earlier the diagnosis, the greater acquisition of skills
Wannenburg and van Niekerk [29]	2018​	To highlight the impact of early intervention in Africa​	Case report​	The tremendous change in Grandin's life after receiving adequate therapy
Osborne et al. [26]	2007​	The association between parental stress and early intervention​	Nonrandomized controlled trial​	Significantly lower levels of parental stress as a consequence of earlier interventions
Malucelli et al. [19]	2021​	Effectiveness of early parental coaching​	Randomized controlled trial​	Parental education on the advantages of early intervention
Rollins et al. [25]	2015​	Community-based early intervention for toddlers with ASD​	Nonrandomized controlled trial​	Beneficial program for attaining appreciable social skills​
Boyd et al. [20]	2018​	Advancing social communication and play program for children with ASD​	Randomized controlled trial​	Classroom-based intervention that helped improve classroom engaging skills in children along with reduced teacher exhaustion
Chamalah and Arsanti [23]	2019​	The role of Quran therapy in children with ASD​	Nonrandomized controlled trial​	A noteworthy outcome in language skills and overall mood​
Karrim et al. [28]	2022​	The role of telerehabilitation services during the COVID-19 pandemic​	Nonrandomized controlled trial​	Online speech therapy sessions as effective as face-to-face sessions
Zhou et al. [24]	2018​	Parent implemented ESDM​	Nonrandomized controlled trial​	The impact of parental education on language and social skills
Gepner et al. [21]	2021​	The effect of slowing down audiovisual information during speech therapy​	Randomized controlled trial​	A favorable outcome in facial recognition and eye fixation
Nair et al. [27]	2021​	Early, low-intensity home-based intervention for children with ASD​	Nonrandomized controlled trial​	A useful intervention for rural, resource-limited areas

Discussion

 *The Role of Early Intervention*

The increasing need for early interventional services for children with ASD continues to rise. It not only has shown promising outcomes but continues to display new advancements. The idea of multiple interventions at once is not necessarily superior, but the time of onset is crucial. ASD can present with different symptoms and can be further classified as mild, moderate, or severe. The primary limitations seen are delayed acquisition of language skills, being socially distant with a preference to be alone, and reduced eye contact. Fortunately, the disorder that was rarely looked into is finally starting to progress. 

Given the aforementioned information, a study by Clark et al. showed children diagnosed early sought immediate treatment options, mainly speech and occupational therapy, while those diagnosed later received aggressive treatment. Even with aggressive treatment, the latter group did not show the same outcome as the former group [30]. Similarly, a study by Rollins et al. showed improved eye contact, verbal reciprocity, and better social skills [25]. Furthermore, a case study by Wannenburg and van Niekerk in Africa emphasized the negative consequences of delaying early intervention. Since Grandin's caretakers lacked knowledge of ASD, she was deprived of speech therapy and remained nonverbal [29]. Communities require more aid, particularly with finances. To no surprise, the financial burden that comes with intensive, early therapy is still a significant hardship that families continue to tackle. Two articles highlight this point and show how a lack of financial support can lead to a delay in starting therapy, which can eventually be distressing [22,28]. However, the study by Karrim et al. on telerehabilitation services for speech therapy during the COVID-19 pandemic brings hope for future families that need effective yet affordable resources [28].

In a home-based study conducted by Nair et al., the implementation of early intervention for children with ASD boosted their expressive language, thereby enhancing their communication skills [27]. Likewise, a one-year clinical trial by Zhou et al. showed a significant difference postintervention in speech and language skills [24]. In a one-month experimental study conducted in Indonesia, Chamalah and Arsanti found a gradual step-up approach critical for displaying successful results [23]. Teachers included in the research study used Quran therapy, starting with Arabic letters and then small chapters to help articulate clear understandable speech. This step-up approach helped children communicate their needs effectively, leading to higher self-esteem [23]. Unfortunately, early intervention services remain scarce in certain parts of the world. A study that calls attention to this point by Alotaibi and Almalki discussed the need for implementing additional resources and the importance of educating communities on the positive outcomes of early intervention [22]. Finally, a common finding was how early intervention did not affect the severity of ASD [24,26,30].

The Effect of Parental Stress

Stress is any change that causes physical, emotional, or psychological strain (WHO). With that in mind, the rising level of stress on parents of children with ASD is an issue that needs extensive attention. An article that allows clear evidence is a clinical trial conducted by Osborne et al. on how parental stress can impact the effectiveness of early interventions. The study revealed children who underwent fewer interventions were from households with higher parental stress. Although some children require time-consuming interventions, this can cause a significant toll on families. On the other hand, a far more significant improvement was seen among children who belonged to households with relatively lower stress levels [26]. During the stressful lockdown of COVID-19, children with ASD received online speech therapy in South Africa as an alternative to face-to-face revealing high parental satisfaction and, eventually, less stress [28].

To understand the possible causes of parental stress, we can explore the link between adequate parental awareness and the magnitude of stress. Early parental coaching has shown to be highly powerful as it can lead to beneficial outcomes. A study conducted by Malucelli et al. exhibits the improvements made in both gross motor and fine motor skills along with higher receptive communication within the study group. Moreover, it displayed better learning abilities, which were astonishing. Contrary to this finding, a decrease in motor skills and learning abilities was noted in the control group. This study shows how early parental education can help parents recognize symptoms, learn how to cope with the diagnosis, and finally improve their overall condition [19].

Realizing parental concern remains just as important as improving the overall outcomes for children with ASD. A crucial step is providing more parental support, whether familial, governmental, or from the community. In the case of Grandin, family support can help parents seek medical aid to bring forth the best care [29]. Not only does stress affect caregivers, but it can also cause a massive impact on teachers, leading to emotional exhaustion and, ultimately, burnout. Boyd et al. demonstrated higher fatigue and burnout levels among teachers who did not receive classroom-based Advancing Social-Communication and Play [20]. In short, the impact of stress can lead to lower quality outcomes in children with ASD coupled with a high psychological burden on caregivers and teachers. 

Understanding the Needs of Children With ASD

The primary goal of prompt intervention is to provide better-quality outcomes for children with ASD. However, to do so, an essential prerequisite is to understand each child individually with a greater perspective. The case report on Grandin allows us to look at the future accomplishments she was able to make after receiving adequate treatment options. Her beautiful relationship with her farm animals made her feel less anxious and improved her well-being [29]. Similarly, a study that helped enhance children's mood was conducted by Chamalah and Arsanti, where implementing Quran therapy helped them feel understood by communicating their needs better and, therefore, a better state of mind. Ultimately, the feeling of being understood can help improve their relationship with peers, leading to a brighter future [23].

It is key to have supportive and loving mentors to be better understood. Commitment to providing children with continuous and effective therapy has shown tremendous results [19,25,26,29,30]. Another essential component is that patience allows children to naturally open up to excel at their own pace [23,29]. When children with autism are perceived differently, the support of good mentors can make a big difference [29]. The influence of teachers on children with ASD can bring about hope for the future. Along with hope, providing children with teachers who are in their best interest can help them flourish.

The result of slowing down can provide multiple beneficial outcomes. Of note, a study conducted by Gepner et al. shows the effect of slowing down audiovisual stimuli on a PC during speech therapy. The clinical trial revealed that children showed better abilities in their vocabulary skills, eye and mouth fixation, and a reduction in inappropriate behavior. Likewise, slowing down certain aspects of their routine can help to a great extent [21]. In contrast, children diagnosed later are given vigorous treatment with the expectation of delivering better outcomes, opposing this aspect. Considering everything, hope for a better future for children with autism remains.

Limitations 

Our systematic review was limited by the number of articles that were included in the final review along with the ratio of randomized versus nonrandomized studies. More extensive studies, especially longitudinal studies should be carried out on this topic to provide additional data. Furthermore, clinical trials need to be stretched out for extended periods to emphasize the benefits of initiating early speech therapy. Additionally, research should include follow-up of patients to demonstrate the advantages of speech therapy comparing the outcomes of affected children who received treatment versus those who did not. That is to say, a five- to 10-year follow-up to inquire about the progress made in their language and communication skills. Research should go beyond the scope of one-on-one therapy with qualified speech therapists and explore the outcomes of group sessions with other affected children. Finally, comorbid conditions such as ADHD should be investigated to identify the role it plays in social communication.

## Conclusions

Based on the studies we reviewed on the effectiveness of initiation of early interventions, mainly speech therapy for children with ASD, it has shown hopeful outcomes for the future. A major improvement in language and communication was prominent, enhancing their social skills among children who underwent earlier intervention. Recognizing the needs of children with ASD can help establish the right treatment plan to provide better and more positive outcomes. Moreover, the treatment’s overall efficacy can further be linked to the stress level among caretakers and teachers, demonstrating a negative correlation. With the cases of ASD substantially increasing, it is crucial to conduct an in-depth study of the disorder to provide answers to questions exploring spontaneous improvement in speech without the need for therapy. As early treatment plans become more common, the benefits and drawbacks should be further explored. Speech therapy is a successful early treatment option to improve chief speech and language skills. Online speech therapy during the COVID-19 pandemic has shown tremendous outcomes and should be considered. This review article highlights the importance of early and regular intervention, suggesting the need to spread awareness beyond the scope it has reached. Short- and long-term goals need to be made independently for each child that should be followed through to yield successful outcomes. 
